# Supplementary material for: Diazinon residues levels in farm-gate Brassica oleracea var. acephala of Kimira-Oluch smallholder farm improvement project, Kenya
Source: PLoS One. 2025 May 28;20(5):e0310586. doi: 10.1371/journal.pone.0310586 (PMC12118883; doi:10.1371/journal.pone.0310586)
Supplement: S1 File — XXX. (PDF) [file pone.0310586.s001.pdf]

S1: Raw Data Farmgate Results

Table of triplicate analyte results for residual diazinon in farm-gate samples of kale from KOSFIP, Kenya (LOQ = 0.01 mg/kg)

Lab Code	Analyte	Results (mg/kg)
AA200065	Diazinon	0.03
AA200066	Diazinon	0.03
AA200067	Diazinon	0.03
AA200068	Diazinon	<LOQ
AA200069	Diazinon	<LOQ
AA200070	Diazinon	0.01
AA200071	Diazinon	0.79
AA200072	Diazinon	0.85
AA200073	Diazinon	0.82
AA200074	Diazinon	0.04
AA200075	Diazinon	0.05
AA200076	Diazinon	0.06
AA200077	Diazinon	1.10
AA200078	Diazinon	1.11
AA200079	Diazinon	0.99
AA200080	Diazinon	0.04
AA200081	Diazinon	0.05
AA200082	Diazinon	0.06
AA200083	Diazinon	0.06
AA200084	Diazinon	0.04
AA200085	Diazinon	0.06
AA200086	Diazinon	0.02
AA200087	Diazinon	0.01
AA200088	Diazinon	0.02
AA200089	Diazinon	<LOQ
AA200090	Diazinon	<LOQ
AA200091	Diazinon	<LOQ
AA200092	Diazinon	0.07
AA200093	Diazinon	0.07
AA200094	Diazinon	0.05
AA200095	Diazinon	0.80
AA200096	Diazinon	0.88
AA200097	Diazinon	0.80
AA200098	Diazinon	0.09
AA200099	Diazinon	0.10

Lab Code	Analyte	Results (mg/kg)
AA200100	Diazinon	0.09
AA200101	Diazinon	0.04
AA200102	Diazinon	0.06
AA200103	Diazinon	0.04
AA200104	Diazinon	0.06
AA200105	Diazinon	0.07
AA200106	Diazinon	0.04
AA200107	Diazinon	0.18
AA200108	Diazinon	0.11
AA200109	Diazinon	0.15
AA200110	Diazinon	0.39
AA200111	Diazinon	0.42
AA200112	Diazinon	0.40
AA200113	Diazinon	0.03
AA200114	Diazinon	0.03
AA200115	Diazinon	0.03
AA200116	Diazinon	0.03
AA200117	Diazinon	0.02
AA200118	Diazinon	0.02
AA200119	Diazinon	0.03
AA200120	Diazinon	0.04
AA200121	Diazinon	0.02
AA200122	Diazinon	0.62
AA200123	Diazinon	0.62
AA200124	Diazinon	0.70
AA200125	Diazinon	0.99
AA200126	Diazinon	0.90
AA200127	Diazinon	0.95
AA200128	Diazinon	0.08
AA200129	Diazinon	0.07
AA200130	Diazinon	0.08
AA200131	Diazinon	0.01
AA200132	Diazinon	0.01
AA200133	Diazinon	0.02
AA200134	Diazinon	0.09
AA200135	Diazinon	0.09
AA200136	Diazinon	0.10
AA200137	Diazinon	0.04
AA200138	Diazinon	0.04
AA200139	Diazinon	0.04
AA200140	Diazinon	0.18

**Lab Code**	**Analyte**	**Results (mg/kg)**
AA200141	Diazinon	0.23
AA200142	Diazinon	0.19
AA200143	Diazinon	0.52
AA200144	Diazinon	0.50
AA200145	Diazinon	0.60
AA200146	Diazinon	<LOQ
AA200147	Diazinon	0.01
AA200148	Diazinon	<LOQ
AA200149	Diazinon	0.05
AA200150	Diazinon	0.05
AA200151	Diazinon	0.05
AA200152	Diazinon	0.14
AA200153	Diazinon	0.11
AA200154	Diazinon	0.10
AA200155	Diazinon	0.04
AA200156	Diazinon	0.05
AA200157	Diazinon	0.04
AA200158	Diazinon	0.65
AA200159	Diazinon	0.61
AA200160	Diazinon	0.70
AA200161	Diazinon	0.43
AA200162	Diazinon	0.41
AA200163	Diazinon	0.49
AA200164	Diazinon	1.06
AA200165	Diazinon	1.10
AA200166	Diazinon	1.01
AA200167	Diazinon	0.05
AA200168	Diazinon	0.06
AA200169	Diazinon	0.07
AA200170	Diazinon	0.72
AA200171	Diazinon	0.71
AA200172	Diazinon	0.80
AA200173	Diazinon	1.21
AA200174	Diazinon	1.00
AA200175	Diazinon	0.79
AA200176	Diazinon	0.04
AA200177	Diazinon	0.05
AA200178	Diazinon	0.04
AA200179	Diazinon	0.43
AA200180	Diazinon	0.40

Lab Code	Analyte	Results (mg/kg)
AA200181	Diazinon	0.41
AA200182	Diazinon	0.57
AA200183	Diazinon	0.61
AA200184	Diazinon	0.68
AA200185	Diazinon	0.05
AA200186	Diazinon	0.05
AA200187	Diazinon	0.03
