# Supplementary material for: Diazinon residues levels in farm-gate Brassica oleracea var. acephala of Kimira-Oluch smallholder farm improvement project, Kenya
Source: PLoS One. 2025 May 28;20(5):e0310586. doi: 10.1371/journal.pone.0310586 (PMC12118883; doi:10.1371/journal.pone.0310586)
Supplement: S2 File — XXX. (PDF) [file pone.0310586.s002.pdf]

S2- Quantitative Analysis Calibration Report

**Batch Data Path**	D:\MassHunter\Data\2020 SAMPLES\March\0303202001\QuantResults\0303202001.batch.bin		
**Analysis Time**	3-4-2020 12:43 PM	**Analyst Name**	admin
**Report Time**	5-6-2020 12:13 PM	**Reporter Name**	admin
**Last Calib Update**	3-4-2020 12:43 PM	**Batch State**	Processed
**Quant Batch Version**	B.05.02	**Quant Report Version**	B.05.02

ISTD Compound ***Dimethoate d6***

Calibration STD	Cal Type	Level	Enabled	Response	RF	RSD	Exp Conc
D:\MassHunter\Data\2020 SAMPLES\March\0303202 001\3.d	Calibration	L1	☑	14949	298.9809		50.0000
D:\MassHunter\Data\2020 SAMPLES\March\0303202 001\46.d	Calibration	L1	☑	14200	284.0026		50.0000
D:\MassHunter\Data\2020 SAMPLES\March\0303202 001\4.d	Calibration	L2	☑	15260	305.2068		50.0000
D:\MassHunter\Data\2020 SAMPLES\March\0303202 001\47.d	Calibration	L2	☑	15406	308.1245		50.0000
D:\MassHunter\Data\2020 SAMPLES\March\0303202 001\5.d	Calibration	L3	☑	14590	291.7904		50.0000
D:\MassHunter\Data\2020 SAMPLES\March\0303202 001\48.d	Calibration	L3	☑	15026	300.5285		50.0000
D:\MassHunter\Data\2020 SAMPLES\March\0303202 001\6.d	Calibration	L4	☑	15627	312.5379		50.0000

Target Compound ***Malathion d10***

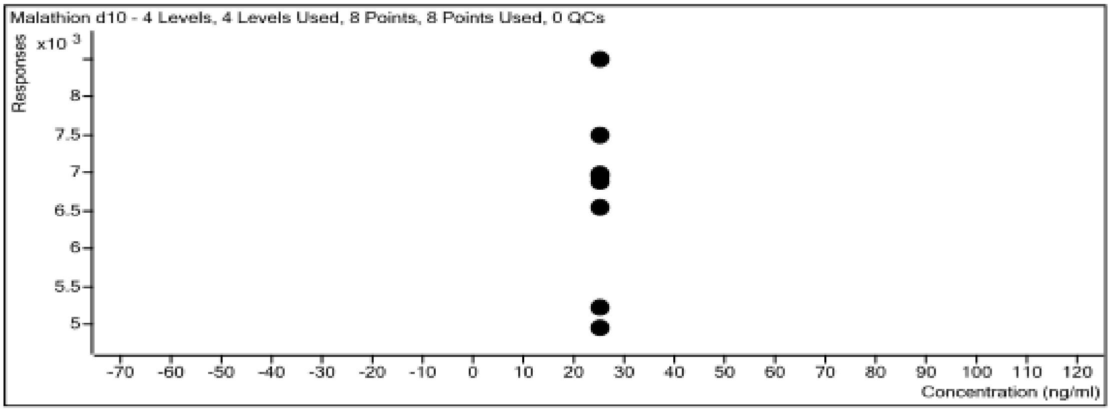

Calibration STD	Cal Type	Level	Enabled	Response	RF	RSD	Exp Conc
D:\MassHunter\Data\2020 SAMPLES\March\0303202 001\3.d	Calibration	L1	☑	8505	340.1822	13.98	25.0000
D:\MassHunter\Data\2020 SAMPLES\March\0303202 001\46.d	Calibration	L1	☑	6975	278.9927	13.98	25.0000
D:\MassHunter\Data\2020 SAMPLES\March\0303202 001\4.d	Calibration	L2	☑	7497	299.8666	6.15	25.0000
D:\MassHunter\Data\2020 SAMPLES\March\0303202 001\47.d	Calibration	L2	☑	6872	274.8772	6.15	25.0000
D:\MassHunter\Data\2020 SAMPLES\March\0303202 001\5.d	Calibration	L3	☑	6946	277.8289	4.32	25.0000
D:\MassHunter\Data\2020 SAMPLES\March\0303202 001\48.d	Calibration	L3	☑	6534	261.3486	4.32	25.0000
D:\MassHunter\Data\2020 SAMPLES\March\0303202 001\6.d	Calibration	L4	☑	5229	209.1418	3.58	25.0000
D:\MassHunter\Data\2020 SAMPLES\March\0303202 001\49.d	Calibration	L4	☑	4970	198.8104	3.58	25.0000

**Batch Data Path**	D:\MassHunter\Data\2020 SAMPLES\March\0303202001\QuantResults\0303202001.batch.bin		
**Analysis Time**	3-4-2020 12:43 PM	**Analyst Name**	admin
**Report Time**	5-6-2020 12:13 PM	**Reporter Name**	admin
**Last Calib Update**	3-4-2020 12:43 PM	**Batch State**	Processed
**Quant Batch Version**	B.05.02	**Quant Report Version**	B.05.02

Target Compound ***Diazinon (Dimpylate)***

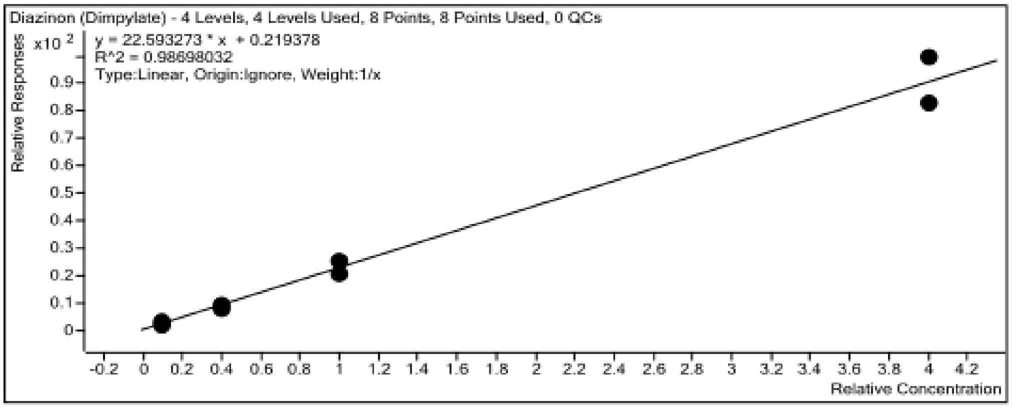

Calibration STD	Cal Type	Level	Enabled	Response	RF	RSD	Exp Conc
D:\MassHunter\Data\2020 SAMPLES\March\0303202 001\3.d	Calibration	L1	☑	30775	20.5867	31.63	5.0000
D:\MassHunter\Data\2020 SAMPLES\March\0303202 001\46.d	Calibration	L1	☑	46078	32.4492	31.63	5.0000
D:\MassHunter\Data\2020 SAMPLES\March\0303202 001\4.d	Calibration	L2	☑	116107	19.0210	14.67	20.0000
D:\MassHunter\Data\2020 SAMPLES\March\0303202 001\47.d	Calibration	L2	☑	144359	23.4255	14.67	20.0000
D:\MassHunter\Data\2020 SAMPLES\March\0303202 001\5.d	Calibration	L3	☑	301337	20.6543	13.67	50.0000
D:\MassHunter\Data\2020 SAMPLES\March\0303202 001\48.d	Calibration	L3	☑	376788	25.0750	13.67	50.0000
D:\MassHunter\Data\2020 SAMPLES\March\0303202 001\6.d	Calibration	L4	☑	1295260	20.7217	12.80	200.0000
D:\MassHunter\Data\2020 SAMPLES\March\0303202 001\49.d	Calibration	L4	☑	1585724	24.8457	12.80	200.0000
