# Supplementary material for: Diazinon residues levels in farm-gate Brassica oleracea var. acephala of Kimira-Oluch smallholder farm improvement project, Kenya
Source: PLoS One. 2025 May 28;20(5):e0310586. doi: 10.1371/journal.pone.0310586 (PMC12118883; doi:10.1371/journal.pone.0310586)

S3: Quality Control Parameters and Selected Chromatograms

1.0 Quality control

a) Raw Co-efficient of Variation (Precision), Repeatability and Recovery Table for six replicates of spikes with three concentrations of 10, 20 and 50 ppb.

		Spiked Levels								
**conc. (ppb)**		Spike 1	Spike 2	Spike 3	Spike 4	Spike 5	Spike 6	C.V	Repeatability	R^2
	a	44.851	41.830	44.258	49.934	52.687	41.883	9.685	4.4256	
**50**	b	43.656	53.799	44.175	54.652	47.712	47.700	9.616	4.5380	0.975
	c	48. 752	46.719	37.334	47.595	48.239	42.331	9.902	4.4384	
	a	-	21.153	19.287	20.102	17.545	18.402	18.325	6.9145	
**20**	b	19.461	17.864	20.071	18.454	19.337	18.968	4.109	1.8559	0.987
	c	18.350	18.529	18.396	17.422	17.781	17.394	2.842	1.5003	
	a	12.078	11.442	11.364	11.084	11.277	13.379	7.280	1.9429	
**10**	b	12.942	12.835	13.209	13.799	11.836	12.522	5.127	1.7041	0.977
	c	12.128	11.741	12.128	12.243	11.854	13.446	4.996	1.6424	

b) Mean Recovery, Coefficient of Variation and Repeatability Units Used in the Calibration Curve of Diazinon Residues in Brassica Oleracea Var. Acephala of KOSFIP

Spiked levels (µg/g)								
	**10**			20			50	
**Rec. (%)**	C.V (%)	Reptty. unit	Rec. (%)	C.V (%)	Reptty. unit	Rec. (%)	C.V (%)	Reptty. unit
**122.958**	5.801	1.7631	93.728	8.426	3.4236	92.623	9.734	4.4673

C.V – Coefficient of Variation; Rec. – Recovery; Reptty. – Repeatability

c) Mean linearity, Limits of Detection (LODs), Limits of Quantification (LOQs) and Maximum Residue Limits (MRLs) used in the calibration curve of diazinon residues in Brassica oleracea Var. acephala of KOSFIP

Linearity		Calibration Limits (µg/g)		MRLs(mg/kg)	
**R^2**	Range(ppb)	LOD	LOQ	Kenya	EU
**0.9797**	5 - 200	10	3.33	0.05	0.01

EU – European Union; LOD – Limits of Detection; LOQ – Limits of Quantitation

2.0 Calibration Curve Chromatograms and Graphics

a) Reagent Blank Chromatogram and Graphics

Analysis Info

**Acq Time**	2020-03-03 15:33	**Data File**	2.d
**Position**	Vial 1	**Sample Name**	reagent blank
**Dilution**	2	**Sample Info**	
**Inj Vol**	-1.00	**Acq Method File**	2308201901 George DIAZINON DMRM.m
**Sample Type**	Sample	**Comment**	

Sample Chromatogram

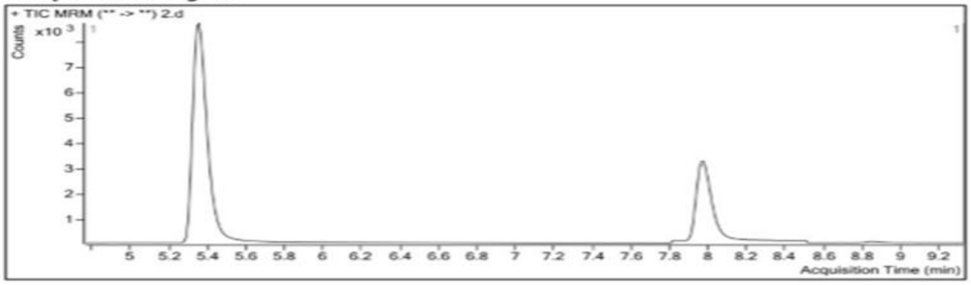

Quantitation Results

Compound	RT	Response	Conc	Accuracy
Malathion d10	7.976	9160		
Diazinon (Dimpylate)	8.852	201	0.0000	

Compound Graphics

ISTD Compound *Dimethoate d6*

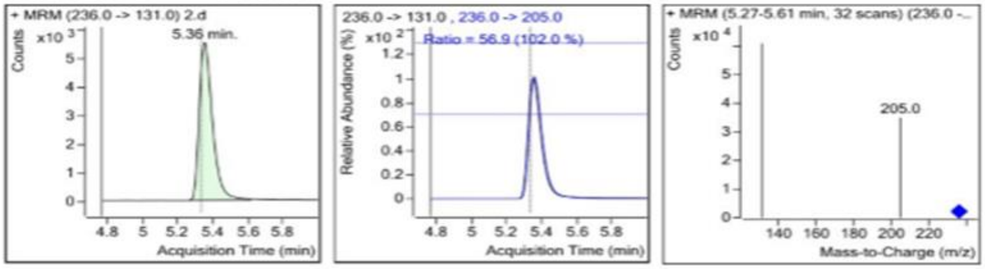

Target Compound *Malathion d10*

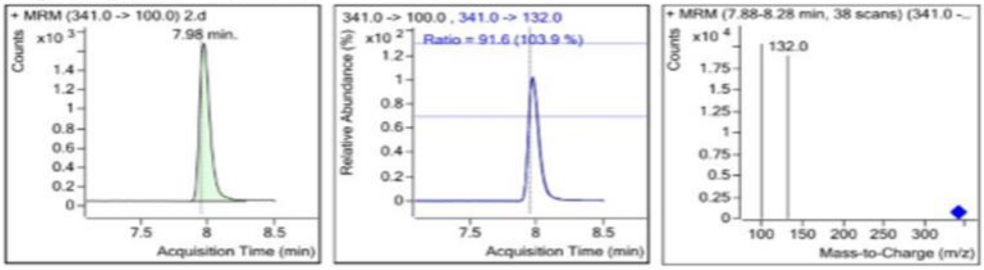

Target Compound *Diazinon (Dimpylate)*

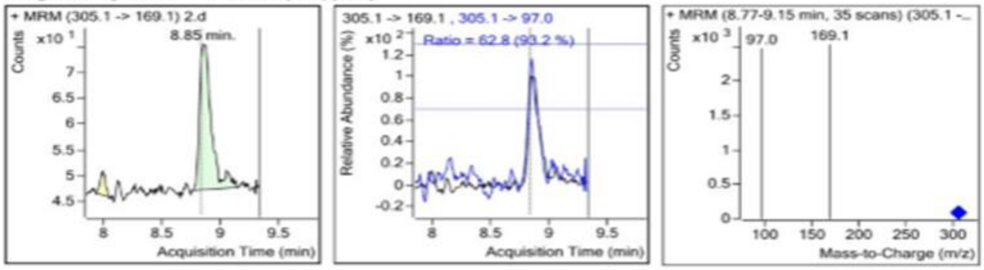

b) 5 ppb Standard Chromatograms and Graphics

Analysis Info

**Acq Time**	2020-03-03 15:47	**Data File**	3.d
**Position**	Vial 2	**Sample Name**	5ppb
**Dilution**	1	**Sample Info**	
**Inj Vol**	-1.00	**Acq Method File**	2308201901 George DIAZINON DMRM.m
**Sample Type**	Calibration	**Comment**	

Sample Chromatogram

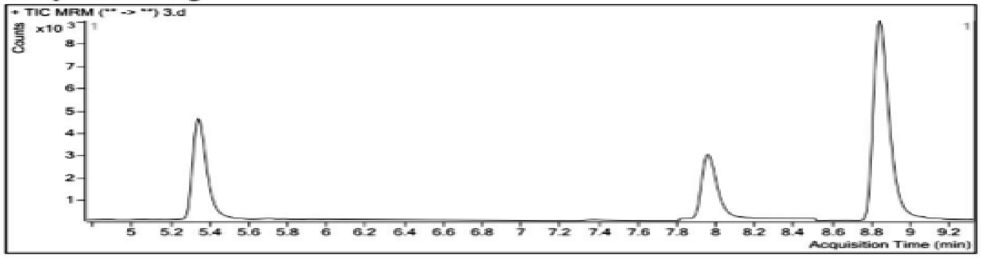

Quantitation Results

Compound	RT	Response	Conc	Accuracy
Malathion d10	7.959	8505		
Diazinon (Dimpylate)	8.844	30775	4.0704	81.41

Compound Graphics

ISTD Compound *Dimethoate d6*

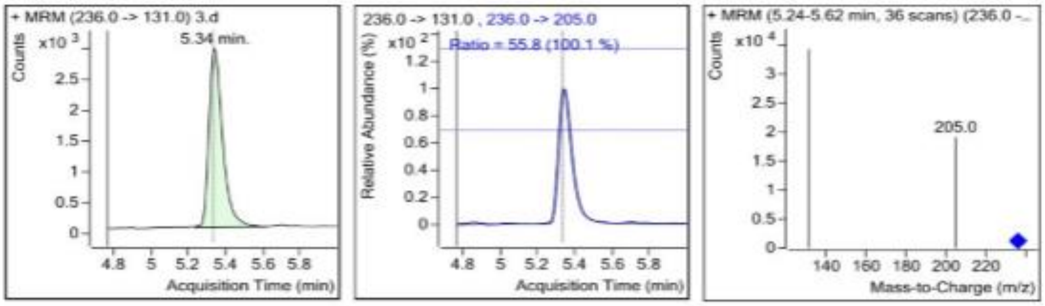

Target Compound *Malathion d10*

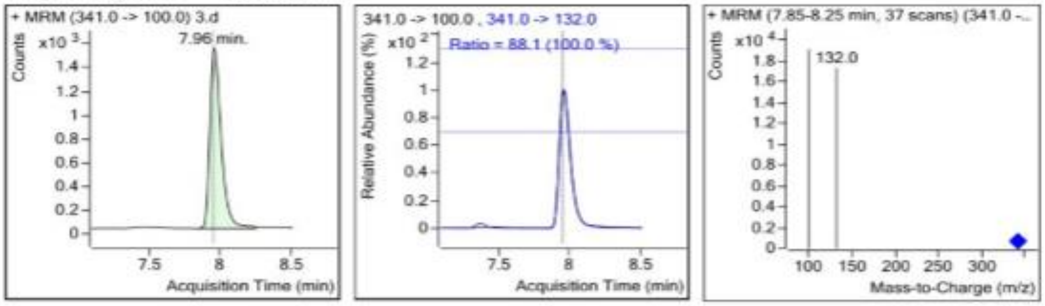

Target Compound *Diazinon (Dimpylate)*

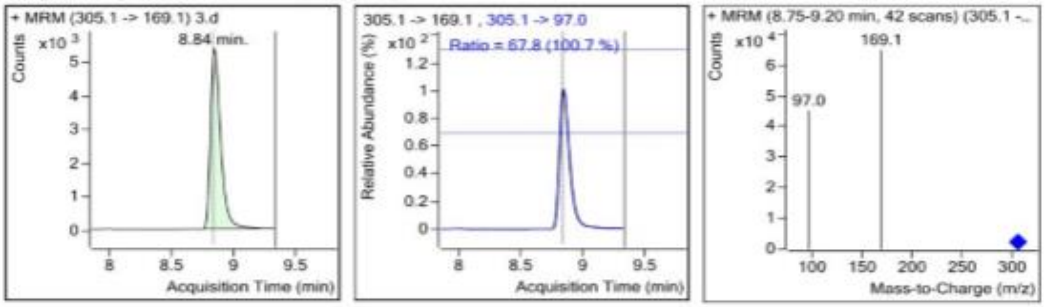

c) **20 ppb Standard Chromatograms and Graphics**

Analysis Info

**Acq Time**	2020-03-03 16:12	**Data File**	4.d
**Position**	Vial 3	**Sample Name**	20ppb
**Dilution**	1	**Sample Info**	
**Inj Vol**	-1.00	**Acq Method File**	2308201901 George DIAZINON DMRM.m
**Sample Type**	Calibration	**Comment**	

Sample Chromatogram

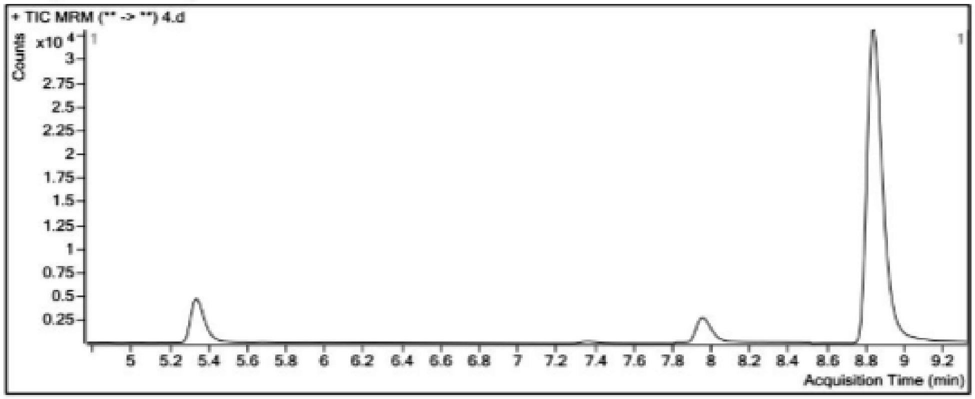

Quantitation Results

Compound	RT	Response	Conc	Accuracy
Malathion d10	7.959	7497		
Diazinon (Dimpylate)	8.844	116107	16.3523	81.76

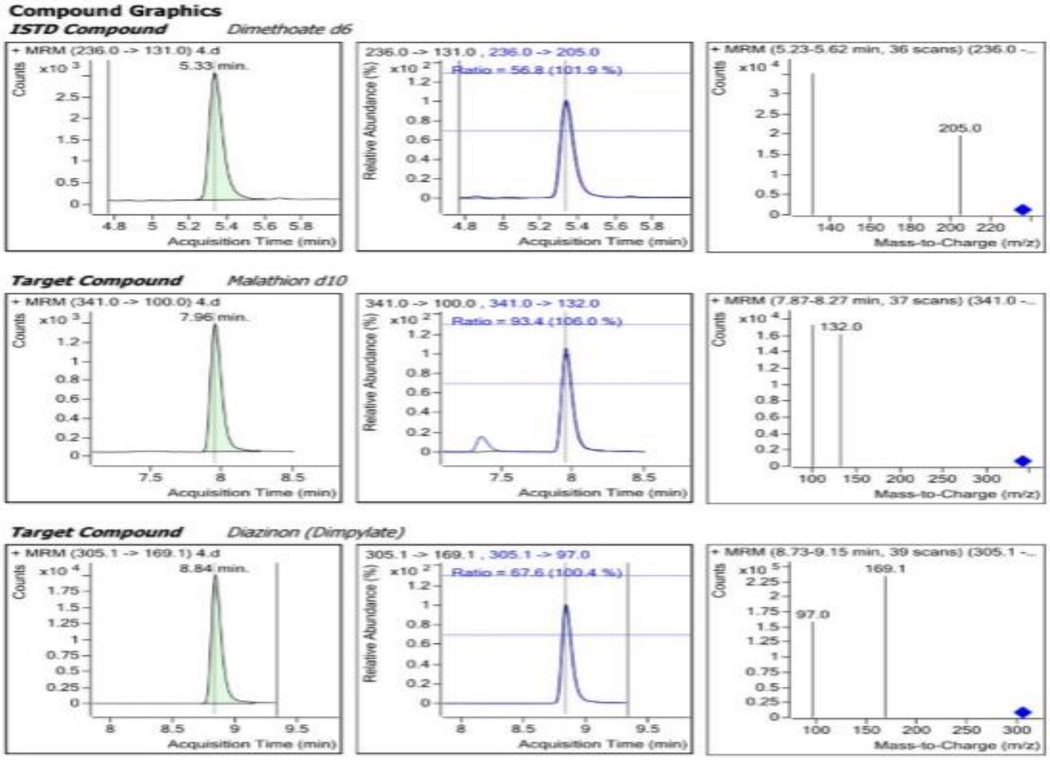

d) **50 ppb Standard Chromatograms and Graphics**

**Batch Data Path**	D:\MassHunter\Data\2020 SAMPLES\March\0303202001\QuantResults\0303202001.batch.bin		
**Analysis Time**	3-4-2020 12:43 PM	**Analyst Name**	admin
**Report Time**	5-7-2020 1:35 PM	**Reporter Name**	admin
**Last Calib Update**	3-4-2020 12:43 PM	**Batch State**	Processed

Analysis Info

**Acq Time**	2020-03-03 16:26	**Data File**	5.d
**Position**	Vial 4	**Sample Name**	50ppb
**Dilution**	1	**Sample Info**	
**Inj Vol**	-1.00	**Acq Method File**	2308201901 George DIAZINON DMRM.m
**Sample Type**	Calibration	**Comment**	

Sample Chromatogram

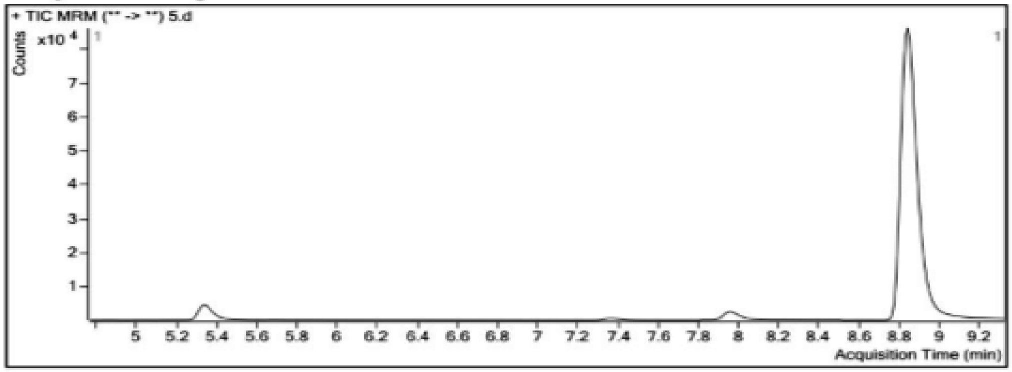

Quantitation Results

Compound	RT	Response	Conc	Accuracy
Malathion d10	7.959	6946		
Diazinon (Dimpylate)	8.844	301337	45.2235	90.45

Compound Graphics

ISTD Compound *Dimethoate d6*

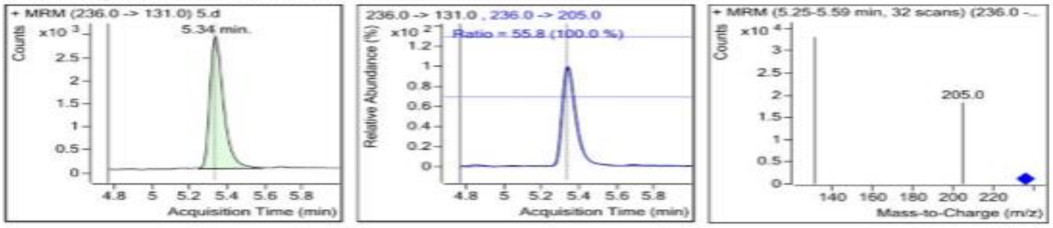

Target Compound *Malathion d10*

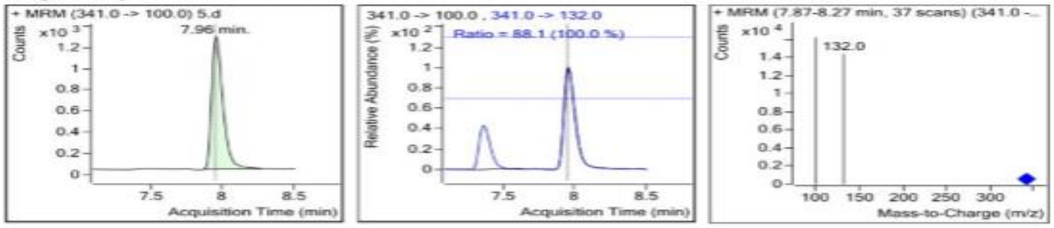

Target Compound *Diazinon (Dimpylate)*

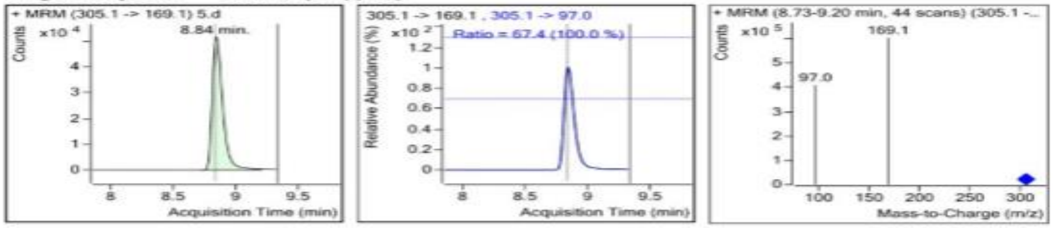

e) 200 ppb Standard Chromatograms and Graphics

Analysis Info

**Acq Time**	2020-03-03 16:28	**Data File**	6.d
**Position**	Vial 5	**Sample Name**	200ppb
**Dilution**	1	**Sample Info**	
**Inj Vol**	-1.00	**Acq Method File**	2308201901 George DIAZINON DMRM.m
**Sample Type**	Calibration	**Comment**	

Sample Chromatogram

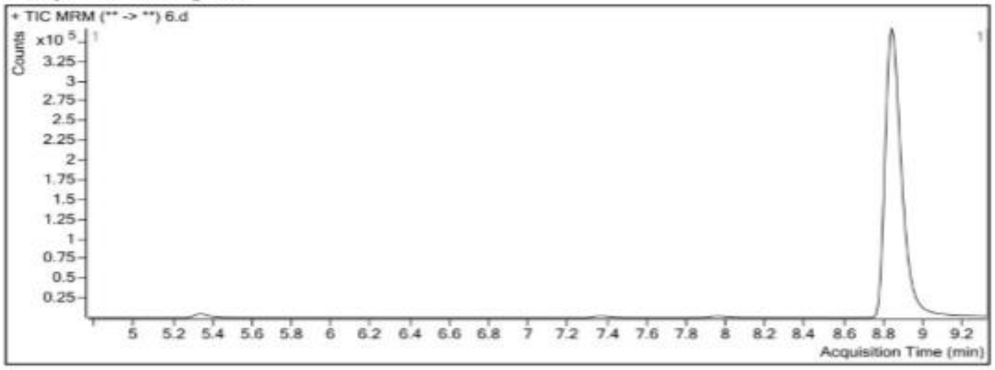

Quantitation Results

Compound	RT	Response	Conc	Accuracy
Malathion d10	7.959	5229		
Diazinon (Dimpylate)	8.844	1295260	182.9465	91.47

Compound Graphics

ISTD Compound *Dimethoate d6*

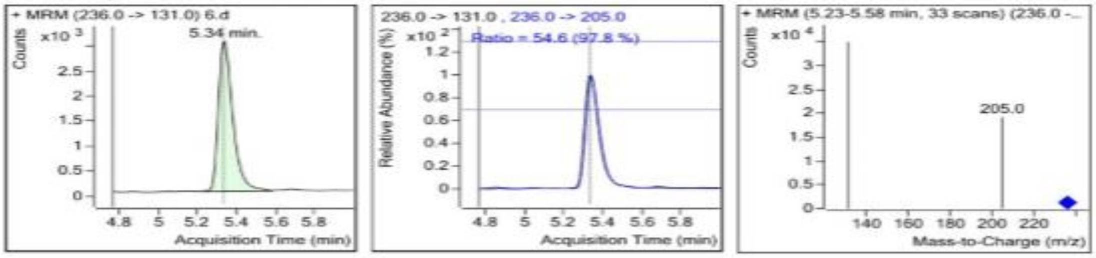

Target Compound *Malathion d10*

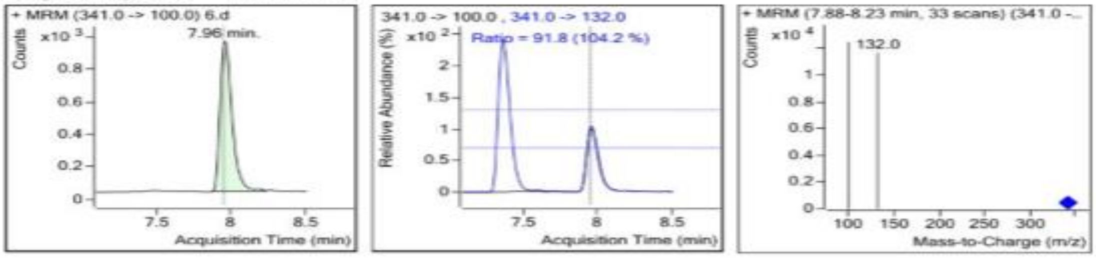

Target Compound *Diazinon (Dimpylate)*

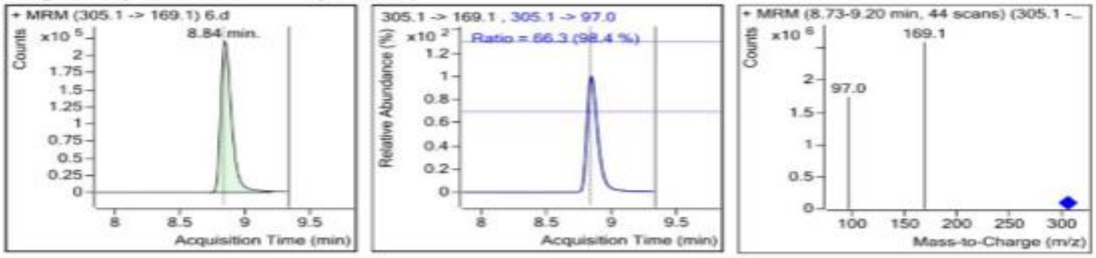

f) Spike Chromatograms and Graphics

Analysis Info

**Acq Time**	2020-03-03 17:07	**Data File**	8.d
**Position**	Vial 7	**Sample Name**	spike
**Dilution**	2	**Sample Info**	
**Inj Vol**	-1.00	**Acq Method File**	2308201901 George DIAZINON DMRM.m
**Sample Type**	Sample	**Comment**	

Sample Chromatogram

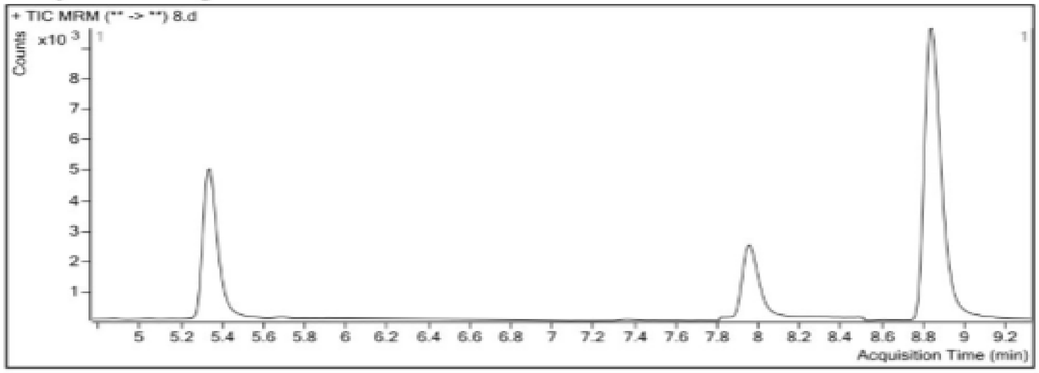

Quantitation Results

Compound	RT	Response	Conc	Accuracy
Malathion d10	7.959	7017		
Diazinon (Dimpylate)	8.844	33545	8.1719	

Compound Graphics

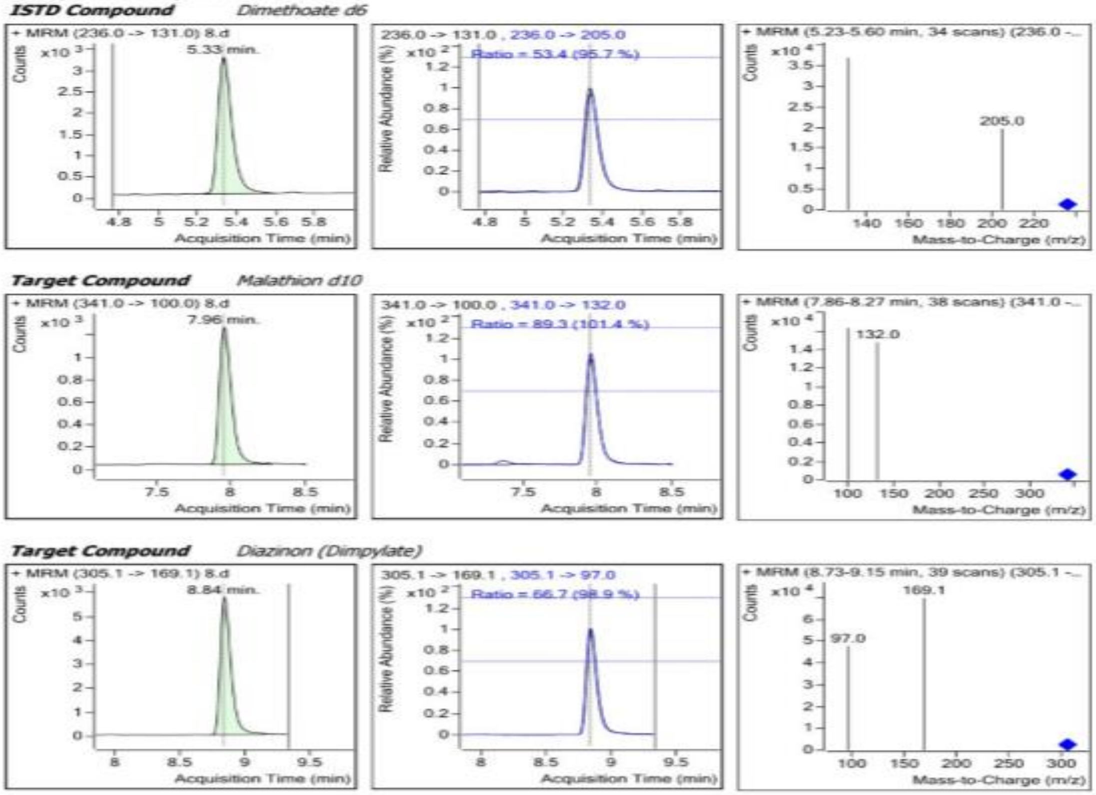

3.0 Calibration Table and Curve for the quantitative determination of analyte in samples

Level	Response	RF	RSD	Expected Conc
L1	30775	20.5867	31.63	5.0000
L1	46078	32.4492	31.63	5.0000
L2	116107	19.0210	14.67	20.0000
L2	144359	23.4255	14.67	20.0000
L3	301337	20.6543	13.67	50.0000
L3	376788	25.0750	13.67	50.0000
L4	1295260	20.7217	12.80	200.0000
L4	1585724	24.8457	12.80	200.0000

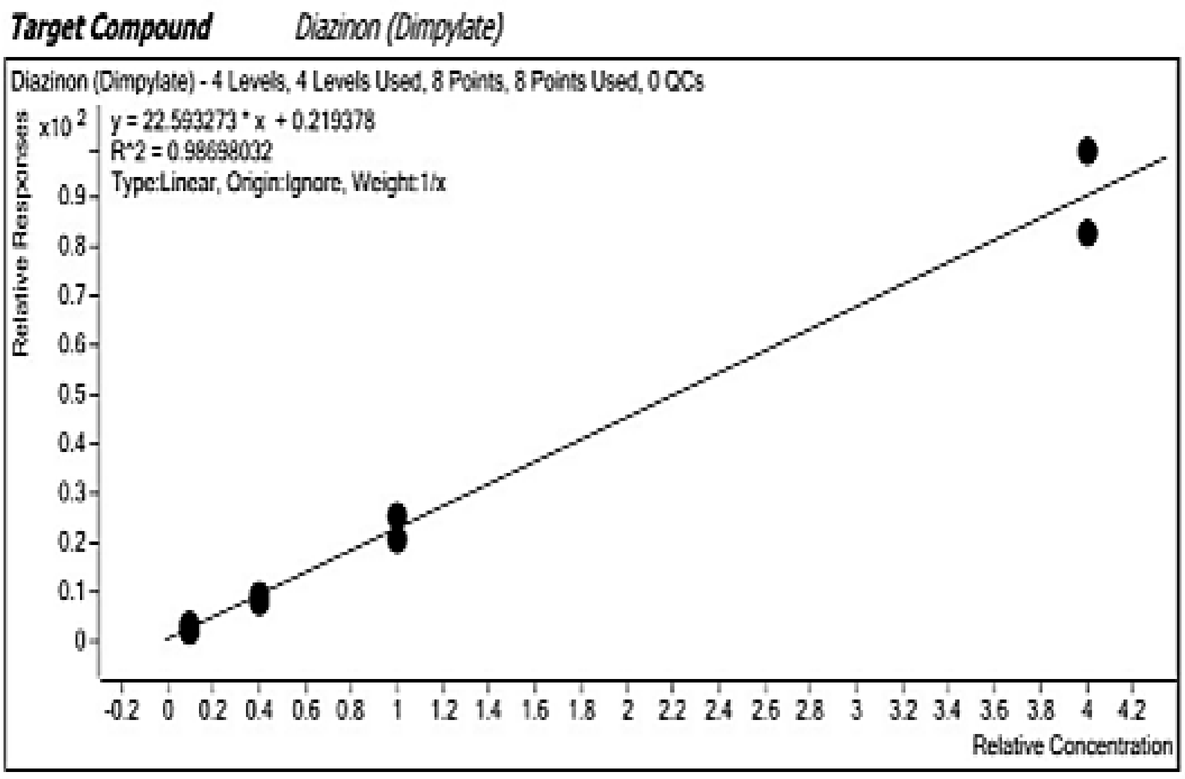

4.0 Selected Sample Chromatograms and Graphics

A. Control sample

Analysis Info

**Acq Time**	2020-03-03 16:53	**Data File**	7.d
**Position**	Vial 6	**Sample Name**	control
**Dilution**	2	**Sample Info**	
**Inj Vol**	-1.00	**Acq Method File**	2308201901 George DIAZINON DMRM.m
**Sample Type**	Sample	**Comment**	

Sample Chromatogram

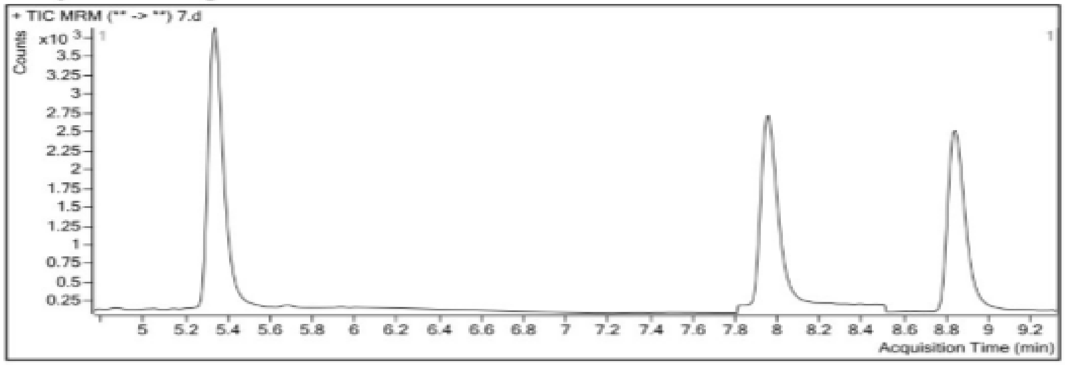

Quantitation Results

Compound	RT	Response	Conc	Accuracy
Malathion d10	7.959	7437		
Diazinon (Dimpylate)	8.844	8427	2.0378	

Compound Graphics

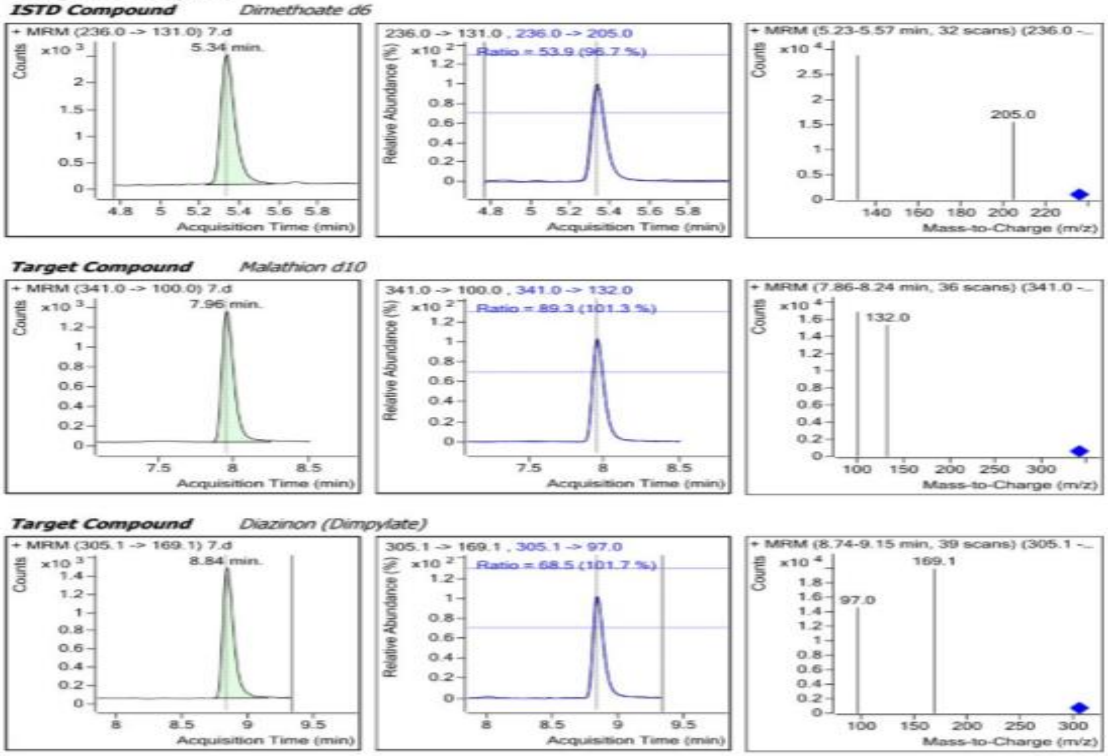

B. i) Selected Sample Chromatograms and Graphics

Analysis Info

**Acq Time**	2020-03-03 17:20	**Data File**	9.d
**Position**	Vial 8	**Sample Name**	AA200461
**Dilution**	2	**Sample Info**	
**Inj Vol**	-1.00	**Acq Method File**	2308201901 George DIAZINON DMRM.m
**Sample Type**	Sample	**Comment**	

Sample Chromatogram

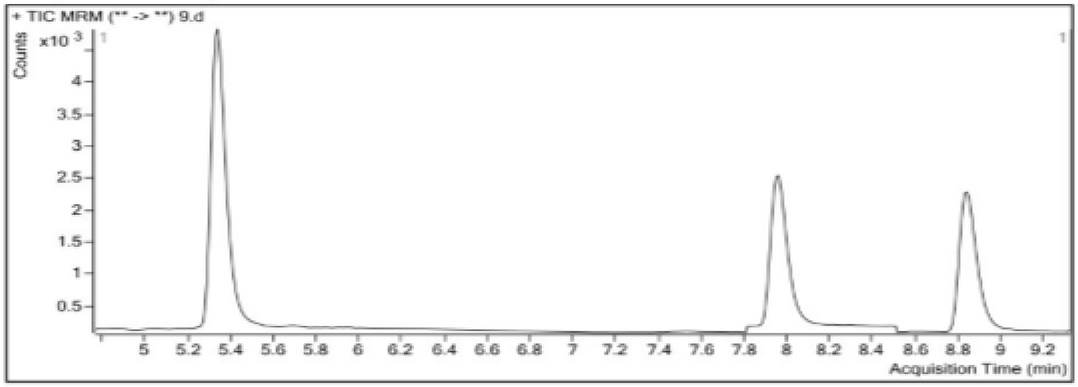

Quantitation Results

Compound	RT	Response	Conc	A[illegible]
Malathion d10	7.959	6860		
Diazinon (Dimpylate)	8.844	7677	1.2106	

Compound Graphics

ISTD Compound *Dimethoate d6*

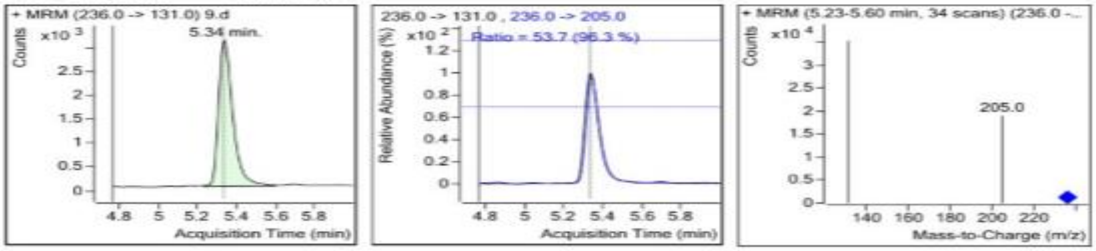

Target Compound *Malathion d10*

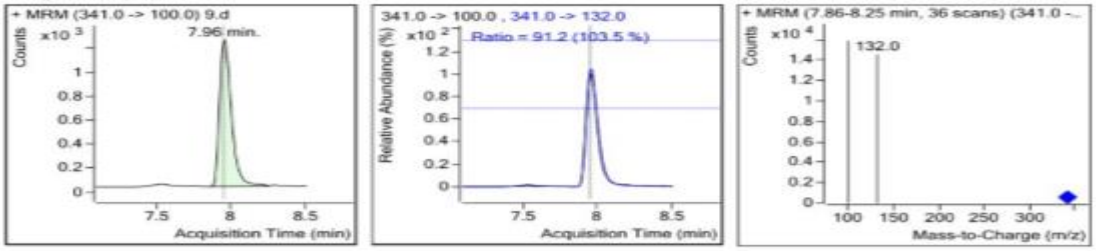

Target Compound *Diazinon (Dimpylate)*

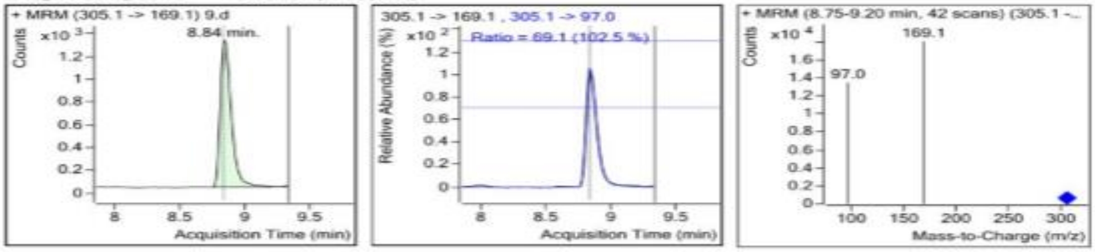

B. ii) Selected Sample Chromatograms and Graphics

Analysis Info

**Acq Time**	2020-03-03 17:34	**Data File**	10.d
**Position**	Vial 9	**Sample Name**	AA200462
**Dilution**	2	**Sample Info**	
**Inj Vol**	-1.00	**Acq Method File**	2308201901 George DIAZINON DMRM.m
**Sample Type**	Sample	**Comment**	

Sample Chromatogram

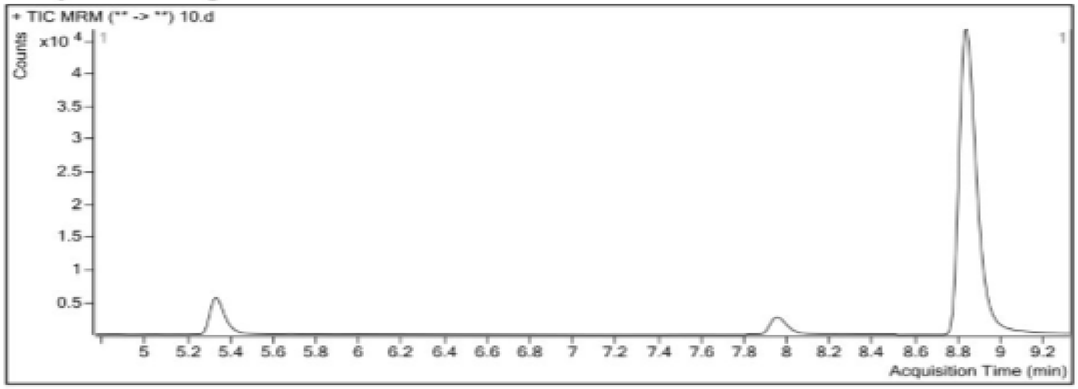

Quantitation Results

Compound	RT	Response	Conc
Malathion d10	7.951	7743	
Diazinon (Dimpylate)	8.844	164335	38.1540

Compound Graphics

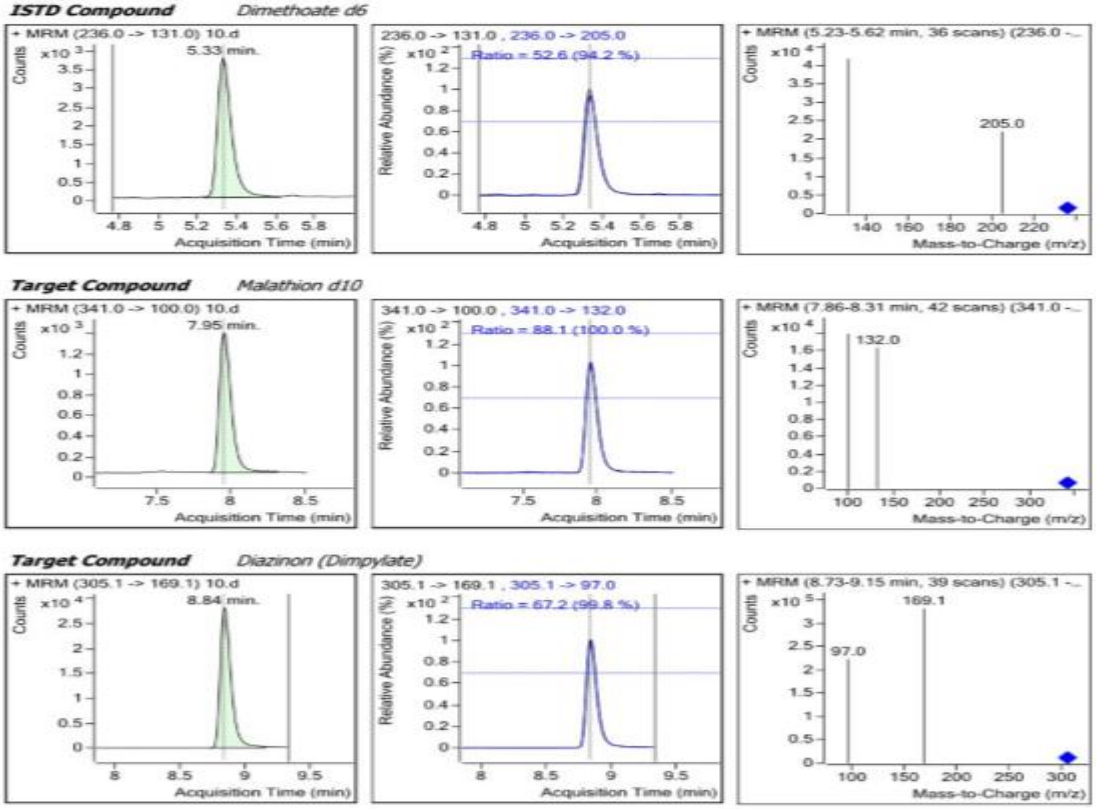

Supplement: S2 File — XXX. (PDF) [file pone.0310586.s003.pdf]
