# Supplementary material for: Diazinon residues levels in farm-gate Brassica oleracea var. acephala of Kimira-Oluch smallholder farm improvement project, Kenya
Source: PLoS One. 2025 May 28;20(5):e0310586. doi: 10.1371/journal.pone.0310586 (PMC12118883; doi:10.1371/journal.pone.0310586)
Supplement: Attachment — Submitted filename: REBUTTAL LETTER.docx [file pone.0310586.s005.docx]

REBUTTAL LETTER

Dear Editor.

In response to PLOS ONE’s decision for the manuscript: “**Diazinon residues levels in farm-gate Brassica oleracea var. Acephala of Kimira-Oluch Smallholder Farm Improvement Project, Kenya”** (Decision: Revision required [PONE-D-24-38603]), we appreciate the Editor’s and reviewer’s comments that have greatly improved this new version of the original research paper.

All the comments and observations have been considered and responded to. The responses are highlighted in the marked-up copy.

a) **Journal Requirements and Editor’s observations**

When submitting your revision, we need you to address these additional requirements.

1. Please ensure that your manuscript meets PLOS ONE's style requirements, including those for file naming. The PLOS ONE style templates can be found at <https://journals.plos.org/plosone/s/file?id=wjVg/PLOSOne_formatting_sample_main_body.pdf>.

and <https://journals.plos.org/plosone/s/file?id=ba62/PLOSOne_formatting_sample_title_authors_affiliations.pdf>.

**Response:** We have ensured that all PLOS ONE manuscript submission requirements including styles are adhered to.

2. Please amend either the title on the online submission form (via Edit Submission) or the title in the manuscript so that they are identical.

**Response:** No amendment was suggested, and we have also not found any reasons to amend the title

3. In your Methods section, please provide additional information regarding the permits you obtained for the work. Please ensure you have included the full name of the authority that approved the field site access and, if no permits were required, a brief statement explaining why.

**Response**: We confirm that no permits were required for the study and methodology. The survey method of sample collection only required informed consent from respondents.

4. We note that your Data Availability Statement is currently as follows: [All relevant data are within the manuscript and its supporting information files]

Please confirm at this time whether or not your submission contains all raw data required to replicate the results of your study. Authors must share the “minimal data set” for their submission. PLOS defines the minimal data set to consist of the data required to replicate all study findings reported in the article, as well as related metadata and methods (<https://journals.plos.org/plosone/s/data-availability#loc-minimal-data-set-definition>. ).

For example, authors should submit the following data:

- The values behind the means, standard deviations and other measures reported;

- The values used to build graphs;

- The points extracted from images for analysis.

Authors do not need to submit their entire data set if only a portion of the data was used in the reported study.

If your submission does not contain these data, please either upload them as Supporting Information files or deposit them to a stable, public repository and provide us with the relevant URLs, DOIs, or accession numbers. For a list of recommended repositories, please see <https://journals.plos.org/plosone/s/recommended-repositories>.

If there are ethical or legal restrictions on sharing a de-identified data set, please explain them in detail (e.g., data contain potentially sensitive information, data are owned by a third-party organization, etc.) and who has imposed them (e.g., an ethics committee). Please also provide contact information for a data access committee, ethics committee, or other institutional body to which data requests may be sent. If data are owned by a third party, please indicate how others may request data access.

**Response:** We have submitted the values behind the means, standard deviation and other measures of dispersion, uploaded as “**Supporting Information files**”. There were no images or graphs. However, the Analytical Chemistry Laboratories (ACL) of Kenya Plant Health and Inspectorate Services (KEPHIS) validated method of extraction is a “restricted property” not to be shared.

5. We notice that your figures are uploaded with the file type 'Other'. Please amend the file type to 'Figure'. Please ensure that each Supporting Information file has a legend listed in the manuscript after the references list.

**Response:** We have amended the file type from ‘Other’ to ‘Figure’

b) **Reviewers Comments and Respective responses from authors**

**Reviewer 1: 1**

In page 4 ,line 118- 136: Extraction and partitioning of samples should be re-written. In this section the authors only needs to mention the extraction and cleanup of the real samples, it is not wise to mention the procedures of addition of standard of diazinon. The addition of standard or the fortification of standard should be mentioned in the preparation of fortified samples in the method validation part. In this section the authors only mention the extraction of diazinon, however, they did not mention the procedures of clean up. It should be addressed properly?

**Action performed:** The extraction and partitioning sections has been rewritten. Statement on diazinon standards and fortification have been transferred to the **method validation section.** However, omission of the clean-up procedure was part of the modifications done in the KEPHIS validated method. The modification provided that since Kale is not a high chlorophyll matrix, omission of clean-up procedure does not affect chromatograms not interfere with instrument quality parameters, and reduces the time and cost of operations.

**Reviewer 1.2**

In page 7, line 172-176: How the authors have measured the mean concentration of Blank sampes in case of LOD and LOQ determination? The determination of LOQ was not appropriate. In case of LOQ determinations please follow the CODEX or EU (SANTE) guidelines.

**Action performed:** The error has been corrected and measurement guidelines provided by EU and Codex have been adopted for use.

**Reviewer 1.3**

In page 8. Iine 200-202: In case of HRI estimation, the ADI value is mentioned for specific pesticides in all food items that the consumers have taken in a day. However the EDI was measured for the specific pesticides in a single food items not for all food items that the consumers have taken in a day, so how it is comparable? Please clarify.

**Action performed:** The computations of HRI have been revised to include partial exposure. The determination of HRI has also been modified to include partial exposure. As a result, the HRI implications have been explained.

**Reviewer 1.4**

In page 12, ine 256: The reference (39) you have cited was an old one, please replace with the new one: Nahar KM., Khan MSI., Habib M., Hossain SM., Prodhan MDH and Islam MA. Health risk assessment of pesticide residues in vegetables collected from northern part of Bangladesh. Food Research 4 (6), 2281-2288.

In page 13, ine 280: The reference (46) you have cited was an old one, please replace with the new one: Habib M., A. Kaium, M.S.I. Khan, M. D. H. Prodhan, N. Begum, M. T. I. Chowdhury, M.A. Islam. 2021. Residue level and health risk assessment of organophosphorus pesticides in eggplant and cauliflower collected from Dhaka city, Bangladesh. Food Research 5 (3), 369-377

The authors are requested to add very recent references in introduction and discussions chapter, you may add the following articles: 1. Multiple pesticide residue determination in major vegetables purchased from Gazipur district of Bangladesh. 2. Analysis of organophosphorus pesticide residues in selected vegetables purchased from Narsingdi district of Bangladesh using QuEChERS Extraction.

**Action performed:** We found the suggested literature quite relevant and included them and a list of other recent publications in the revised document.

**Reviewer 2:1**

Abstract: It should be concise with quantified data and more information about Diazinon and its uses in Brassica. The research implications are very wide. Please give the precise implications of the study.

**Action performed:** The abstract has been rewritten in a precise manner with quantified data and additional information on the use of diazinon in Brassica. The research implications have been made more specific

**Reviewer 2:2**

Introduction: The sentences “These conditions promote the rapid spread of vegetable pests and diseases (20,21). The smallholder farmers manage the pests by use of synthetic pesticides.” are very broad and irrelevant to study. Please give specific mentions of insect pests and control by which insects in Brassica field in particular locations recommended by the authority.

**Action performed:** The section has been rewritten specifying a list of pests that are known to infest Kale. An explanation has been provided as to how Diazinon controls and eradicates insect pests.

**Reviewer 2:3**

No scientific hypothesis and research gaps are provided by the authors in the introduction part.

**Action performed:** Scientific hypothesis and research gaps have been stated succinctly and highlighted

**Reviewer 2:4**

“Brassica oleracea var. acephala” once it has been used; after that, it gives a short name for this.

**Action performed:** The botanical name of the study vegetable**,** *Brassica oleracea* var. *acephala* has been used and its English equivalent name *Kale* is used consistently throughout the document.

**Reviewer 2:5**

Separate result and discussion part

**Action performed:** Results and discussions sections have been separated

**Reviewer 2:6**

Line 235-236: “The data set (Table 1) had no outliers”. What was the method of outlier detection?

**Action performed:** We calculated minimum and maximum outliers based on the interquartile range method formula for outlier detection-

i) Minimum outlier = Q1-1.5 (IQR)
ii) Maximum outlier = Q3+1.5(IQR)

Where Q is quartile; IQR is interquartile range

From the calculations, the minimum and maximum outlier values were outside the range of residual values obtained from the instrument readings. We therefore concluded that there was no outlier.

**Reviewer 2:7**

Calibration, validation and peak of pesticide detection are to be provided for review purposes.

**Action performed:** Calibration curve and peak of pesticide detection have been provided herein as Figure 3. Few chromatograms have been listed under ‘Supporting Information File’.

**Reviewer 2:8**

Specific recommendations and utility of the present research at the end of the discussion and conclusion are to be provided in the revised manuscript.

**Action performed:** We have rewritten the specific recommendation and utility of the research to make them more specific and relevant to the problem of study.

Yours sincerely,

George O. Oromo

(**Corresponding author**)
